# Inhibition of HSF1 suppresses the growth of hepatocarcinoma cell lines *in vitro* and AKT-driven hepatocarcinogenesis in mice

**DOI:** 10.18632/oncotarget.16927

**Published:** 2017-04-07

**Authors:** Antonio Cigliano, Chunmei Wang, Maria G. Pilo, Marta Szydlowska, Stefania Brozzetti, Gavinella Latte, Giovanni M. Pes, Rosa M. Pascale, Maria A. Seddaiu, Gianpaolo Vidili, Silvia Ribback, Frank Dombrowski, Matthias Evert, Xin Chen, Diego F. Calvisi

**Affiliations:** ^1^ Institut für Pathologie, Universitätsmedizin Greifswald, Greifswald, Germany; ^2^ Department of Bioengineering and Therapeutic Sciences and Liver Center, University of California, San Francisco, CA, USA; ^3^ Department of Clinical and Experimental Medicine, University of Sassari, Sassari, Italy; ^4^ Pietro Valdoni Surgery Department, University of Rome La Sapienza, Rome, Italy; ^5^ Institut für Pathologie, Universitätsklinikum Regensburg, Regensburg, Germany

**Keywords:** hepatocellular carcinoma, HSF1, signaling pathways

## Abstract

Upregulation of the heat shock transcription factor 1 (HSF1) has been described as a frequent event in many cancer types, but its oncogenic role in hepatocellular carcinoma (HCC) remains poorly delineated. In the present study, we assessed the function(s) of HSF1 in hepatocarcinogenesis via *in vitro* and *in vivo* approaches. In particular, we determined the importance of HSF1 on v-Akt murine thymoma viral oncogene homolog (AKT)-induced liver cancer development in mice. We found that knockdown of HSF1 activity via specific siRNA triggered growth restraint by suppressing cell proliferation and inducing massive cell apoptosis in human HCC cell lines. At the molecular level, HSF1 inhibition was accompanied by downregulation of the phosphoinositide 3-kinase (PI3K)/AKT/mammalian target of rapamycin (mTOR) cascade and related metabolic pathways. Most importantly, overexpression of a dominant negative form of HSF1 (HSF1dn) in the mouse liver via hydrodynamic gene delivery led to the inhibition of mouse hepatocarcinogenesis driven by overexpression of AKT. In human liver cancer specimens, we detected that HSF1 is progressively induced from human non-tumorous surrounding livers to HCC, reaching the highest expression in the tumors characterized by the poorest outcome (as defined by the length of patients’ survival). In conclusion, HSF1 is an independent prognostic factor in liver cancer and might represent an innovative therapeutic target in HCC subsets characterized by activation of the AKT/mTOR pathway.

## INTRODUCTION

Hepatocellular carcinoma (HCC) is rated as the sixth most frequent tumor and the second most common cause of cancer-related mortality, with ˜745,000 deaths worldwide. Although potentially curative modalities such as liver resection, liver transplantation, and radiofrequency ablation, have been developed [1, 2], most HCC patients are diagnosed at advanced stage, when these effective treatments cannot be applied [1, 2]. The only approved drug for the treatment of advanced HCC by the American Food and Drug Administration is the multikinase inhibitor Sorafenib. However, based on large clinical trials conducted in Caucasians and Asians, the improvement in terms of patient's survival following Sorafenib administration is limited to ˜3 months when compared to placebo [3]. Therefore, novel therapeutic strategies against advanced HCC are urgently needed. For this purpose, a more profound understanding of the molecular mechanisms underlying hepatocarcinogenesis is highly required.

Heat shock factor 1 (HSF1) is a member of the heat shock protein (HSP) family, which can differentially regulate the expression of numerous proteins in response to a variety of stressors [4–9]. Once activated, HSF1 implements a versatile series of events aimed at protecting cells from damage induced by continual exposure to the initial insult [4–9]. In addition to orchestrating the heat shock response, HSF1 is involved in numerous other cellular processes, as recent studies have shown that HSF1 regulates as much as 3% of the entire genome [10]. In accordance with this hypothesis, it has been shown that HSF1 is involved in various cellular events, such as metabolism, inflammation, and tumorigenesis as well as in the protection of the cell by numerous stressors beyond the heat shock [11–14]. Although activation of HSF1 might be beneficial for the survival of normal cells, it is plausible to hypothesize that its aberrant activation plays a role in carcinogenesis by protecting tumor cells from stress-induced death. In accordance with this assumption, many human tumor types and cancer cell lines express HSF1 constitutively at elevated levels [11–16]. In cancer, it has been shown that HSF1 could not be regarded as a canonical oncogene, as its overexpression cannot transform immortalized mouse embryonic fibroblasts (MEFs) [17]. Nonetheless, it has been demonstrated that MEFs deprived of HSF1 are refractory to transformation induced by established protooncogenes [17]. In agreement with this finding, HSF1 knockout (HSF1^−/−^) mice were found to be significantly more resistant to tumor formation than wild-type mice in different models of chemically-induced carcinogenesis [17]. Furthermore, deficiency of HSF1 prevented tumor formation in mice harboring either germline mutations in the p53 tumor suppressor or activating mutations in the H-Ras oncogene [17]. In addition, loss of HSF1 in a p53-deficient mouse model resulted in the suppression of spontaneous lymphoma development [18].

In liver cancer, data on HSF1 are rather scanty. Recent findings point to a role of HSF1 as a key determinant of HCC development by its ability to promote hepatic steatosis and to inhibit AMP-activated protein kinase activity [19]. Moreover, HSF1 upregulation is involved in invasion and metastasis and might represent a prognostic marker in HCC [20, 21]. In addition, suppression of HSF1 inhibits the epithelial-mesenchymal transition phenotype in HCC cell lines [22] and protects HCC cells from DNA damage [23]. Furthermore, HSF1 has been found to be a downstream effector of the mammalian target of rapamycin (mTOR) pathway in this tumor type [24]. However, the signalling pathways responsible for HSF1 activation and downstream effectors of HSF1 in HCC cells remain poorly delineated. Also, the importance of HSF1 on hepatocarcinogenesis driven by established oncogenes has never been demonstrated *in vivo*.

In the present study, we found that HSF1 regulates the PI3K/AKT/mTOR pathway in HCC cells and is required for hepatocarcinogenesis triggered by AKT overexpression. Furthermore, we provide evidence that HSF1 is an independent prognostic factor for human HCC.

## RESULTS

### Suppression of HSF1 reduces the *in vitro* growth and AKT/mTOR activity of human HCC cell lines

**Figure 1:**
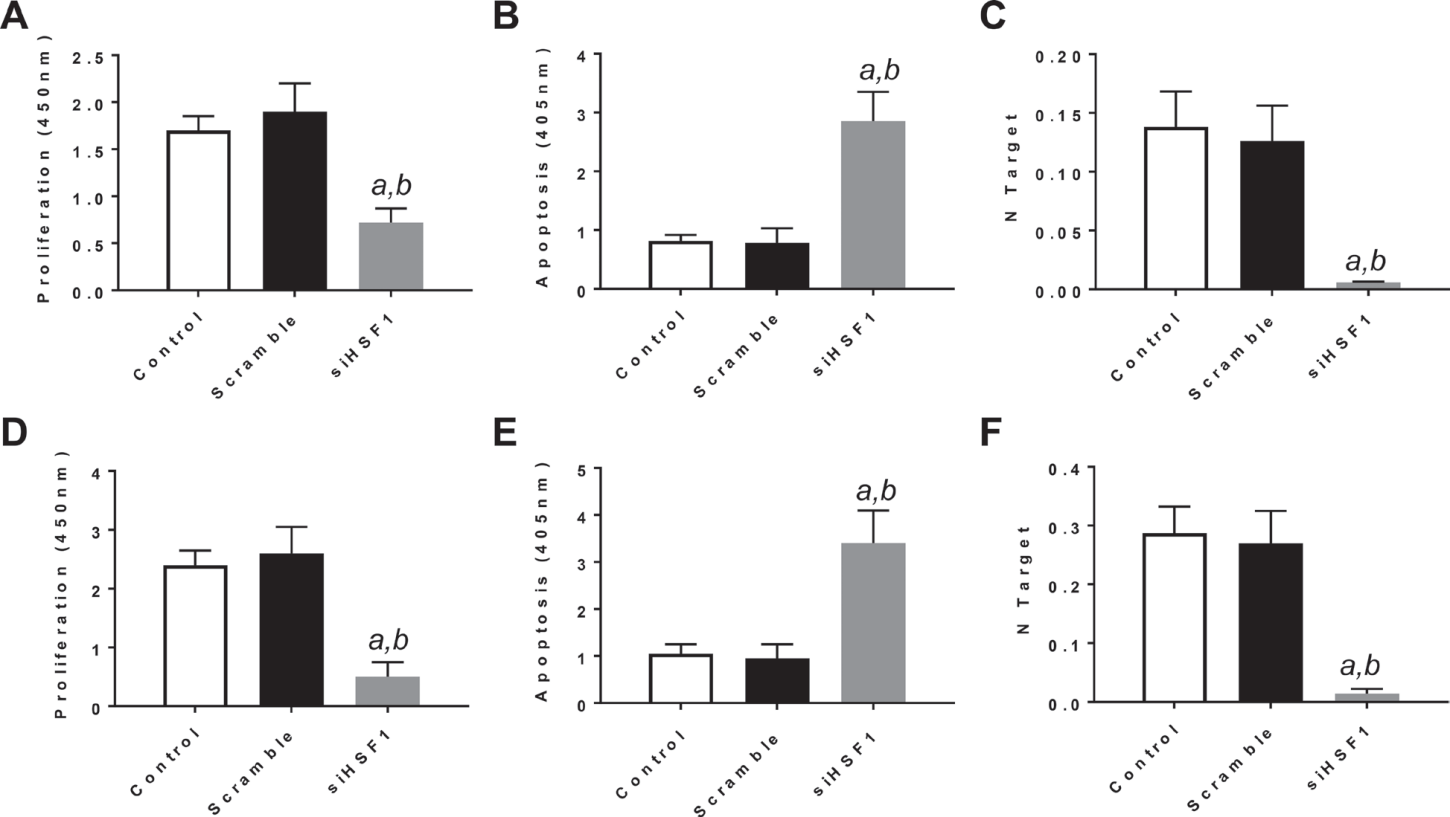
Inactivation of HSF1 is detrimental for the *in vitro* growth of human HCC cell lines (**A**–**C**) Effect of *HSF1* silencing via siRNA in HLE cells on cell proliferation (A), apoptosis (B), and *HSF1* mRNA expression (C). (**D**–**F**) Effect of *HSF1* silencing via siRNA in HLF cells on cell proliferation (D), apoptosis (E), and *HSF1* mRNA expression (F). Each *bar* represents mean ± standard deviation of 3 independent experiments conducted in triplicate. Effects at 48h post siRNA administration are shown. Number target (NT) = 2^−ΔCt^, wherein the ΔCt value of each sample was calculated by subtracting the average Ct value of the *HSF1* gene from the average Ct value of the β-actin gene. Tukey–Kramer test: *P* < 0.0001 *a*, vs control (untreated cells); *b*, vs scramble siRNA.

First, we assessed the importance of HSF1 on the *in vitro* growth and the relationship with the AKT/mTOR pathway in human HCC cell lines. For this purpose, the *HSF1* gene expression was knocked-down in HLE and HLF hepatoma cells with specific small interfering RNA (siRNA) (Figure 1). Knockdown of *HSF1* by siRNA resulted in strong reduction of proliferation and increase of apoptosis in the two cell lines, implying a pivotal role for HSF1 in the growth and survival of HCC cells (Figure 1).

**Figure 2:**
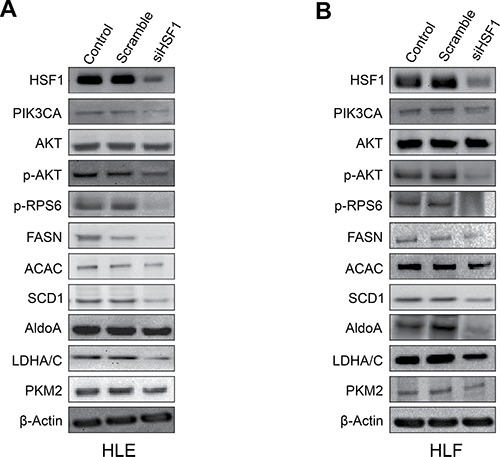
Suppression of *HSF1* expression by specific siRNA induces downregulation of the PI3K/AKT/mTOR pathway (**A**) In HLE cells, silencing of HSF1 for 48 h resulted in the downregulation of PIK3CA (an upstream inducer of AKT), phosphorylated/activated AKT and downstream AKT effectors involved in lipogenesis (p-RPS6, FASN, ACAC, SCD1) and glycolysis (ALDOA, LDHA/C, PKM2), as detected by Western blot analysis. (**B**) Equivalent results were obtained in HLF cells. β-Actin was used as a loading control.

**Figure 3:**
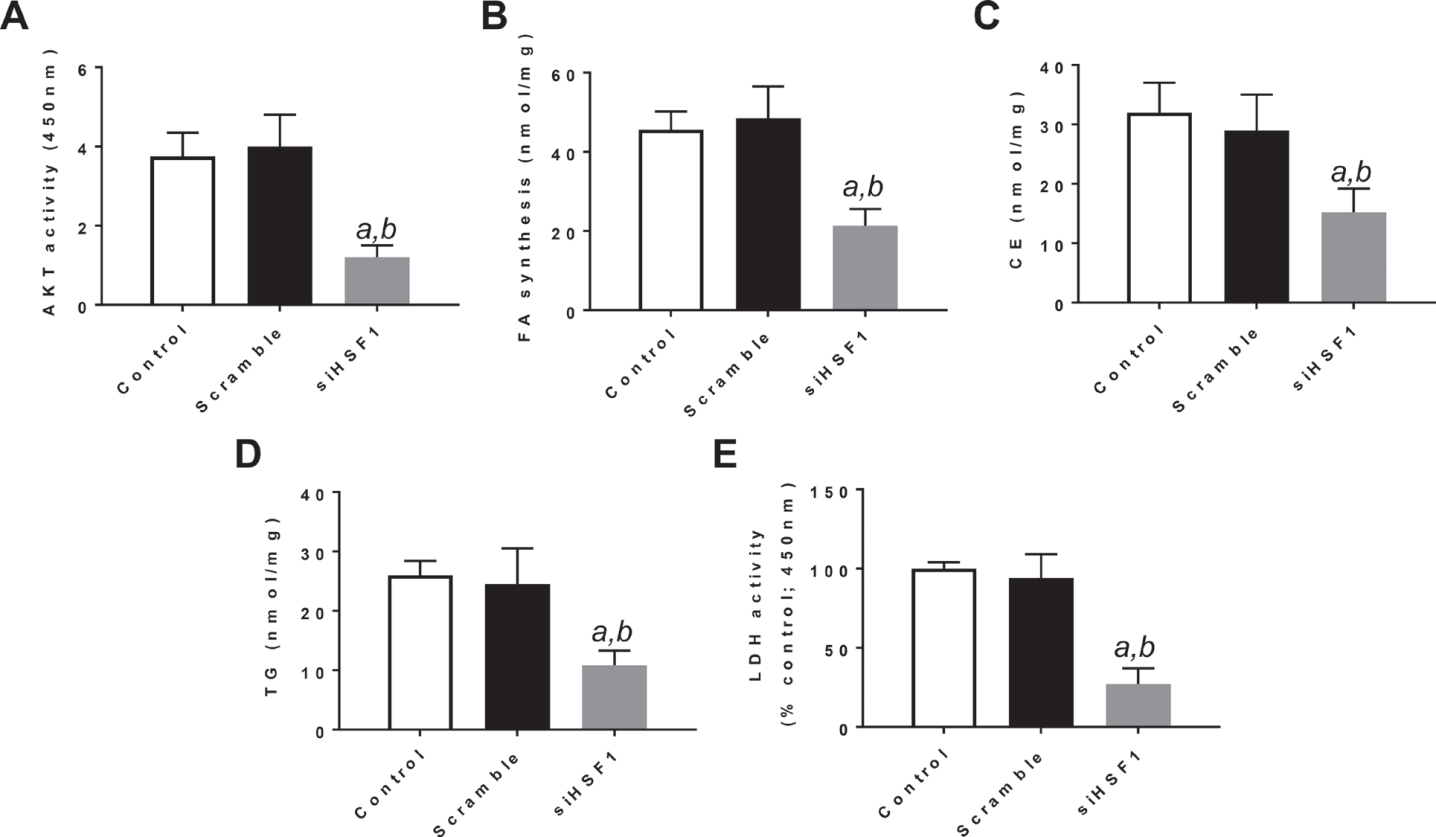
Suppression of *HSF1* expression by specific siRNA induces decrease in AKT activity, fatty acid biosynthesis, cholesterol and triglyceride levels, and lactate dehydrogenase activity in the HLE HCC cell line Equivalent results were obtained in the HLF cell line (Supplementary Figure 2). Each *bar* represents mean ± standard deviation of 3 independent experiments conducted in triplicate. Tukey–Kramer test: *P* < 0.0001 *a*, vs control (untreated cells); *b*, vs scramble siRNA. Abbreviations: FA, fatty acid; CE, cholesterol; TG, triglycerides; LDH, lactate dehydrogenase.

Next, since it has been shown that HSF1 can induce the activation of the PI3K/AKT/mTOR cascade [19], we investigated the crosstalk between HSF1 and the AKT/mTOR cascade *in vitro*. For this purpose, we determined whether HSF1 modulation affects the levels of the PI3K/AKT/mTOR pathway. Noticeably, inhibition of *HSF1* by siRNA led to the downregulation of various members of the PI3K/AKT/mTOR cascade. At the molecular level, HLE and HLF cells depleted of HSF1 showed decreased levels of phosphatidylinositol-4,5-bisphosphate 3-kinase catalytic subunit alpha (PIK3CA), phosphorylated/activated AKT or p-AKT, phosphorylated/activated ribosomal protein S6 or p-RPS6, members of AKT-dependent lipogenesis such as fatty acid synthase or FASN, acetyl-coA carboxylase or ACAC, and stearoyl-CoA desaturase or SCD1. Members of the glycolysis pathway driven by the PI3K/AKT/mTOR cascade, including aldolase A or ALDOA, lactate dehydrogenase A/C or LDHA/C, and pyruvate kinase M2 (PKM2) were also downregulated following HSF1 inactivation (Figure 2). Suppression of HSF1 also resulted in decreased AKT activity, lipid depletion (as indicated by reduced levels of fatty acid biosynthesis, cholesterol and triglycerides), and decrease glycolysis (as shown by decreased activity of lactate dehydrogenase) (Figure 3; Supplementary Figure 1).

The present data indicate that inhibition of HSF1 is highly harmful for the *in vitro* growth of HCC cells, and HSF1 is a major regulator of the PI3K/AKT/mTOR signaling in human HCCs.

### Inactivation of HSF1 suppresses AKT-driven hepatocarcinogenesis

**Figure 4:**
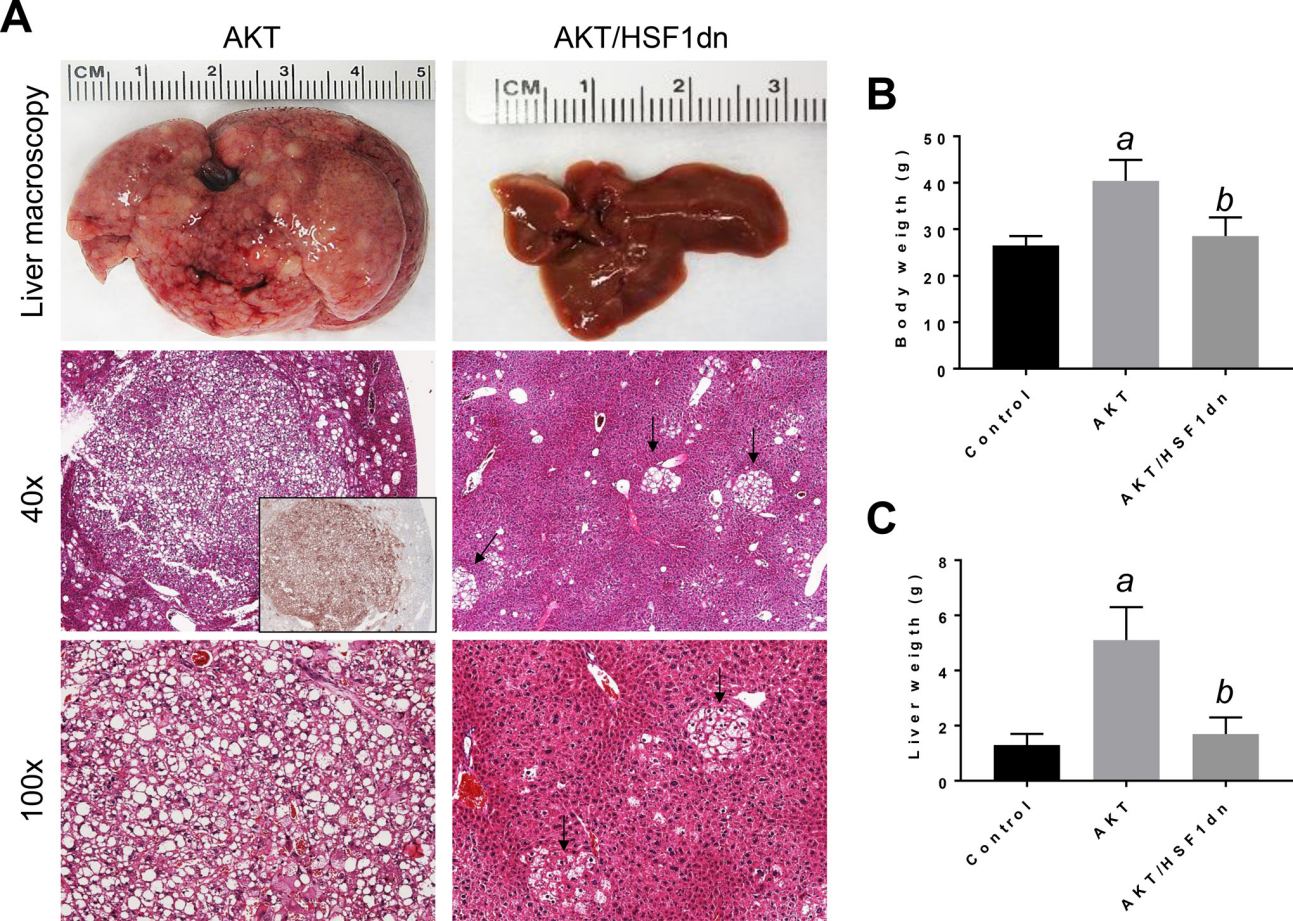
Inactivation of HSF1 abolishes AKT-driven hepatocarcinogenesis in mice (**A** left panel). Overexpression of *myr-AKT1* promotes the development of multiple liver tumors within 28 weeks post hydrodynamic injection in mice (indicated as AKT). Macroscopically (liver macroscopy), liver of AKT mice appeared pale, enlarged, and characterized by the presence of numerous nodules occupying most of its surface (*top* left panel). Microscopically, the liver parenchyma of AKT mice was occupied by numerous hepatocellular tumors (middle and lower left panel). These tumors were homogeneously immunoreactive for HA-tagged AKT (*inset*), implying their origin from hydrodynamically transfected cells. Hepatocellular tumors were mainly composed of malignant cells with an enlarged, clear cytoplasm owing to lipid accumulation (lower panel). In striking contrast, overexpression of a dominant negative form of HSF1 (*HSF1dn*) together with *myr-AKT1* (indicated as AKT/HSF1dn mice) completely inhibits hepatocarcinogenesis (right panels). Livers of AKT/HSF1dn mice appeared normal macroscopically, and showed the presence of few foci, consisting of lipid rich hepatocytes (indicated by arrows). Original magnifications: 40X and 100x. Inactivation of HSF1 by injection of *HSF1dn* also resulted in lower body weight (**B**) and lower liver weight (**C**) in AKT/HSF1dn mice when compared with AKT mice. Tukey–Kramer test: *P* < 0.0001 *a*, vs control (mice injected with empty vector); *b*, vs AKT mice. Ten mice per each group were analyzed. Abbreviation: H&E, hematoxylin and eosin.

**Figure 5:**
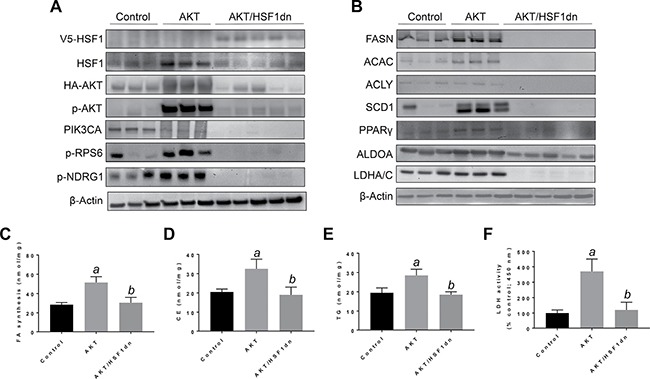
Suppression of hepatocarcinogenesis following HSF1 inactivation is accompanied by downregulation of activated AKT and its downstream effectors in the mouse liver (**A**, **B**) Representative Western blot analysis of livers from control mice (injected with empty vector), AKT mice (injected with *myr-AKT1*) and AKT/HSF1dn mice (co-injected with *myr-AKT1* and *HSF1dn*). Inactivation of HSF1 in AKT/HSF1dn mice resulted in downregulation of phosphorylated/activated AKT. Levels of some AKT upstream (PIK3CA) and downstream effectors (p-RPS6, p-NDRG1, FASN, ACLY, ACAC, SCD1, PPARG, ALDOA, LDHA/C, PKM2) were downregulated in AKT/HSF1dn mice when compared with AKT mice. As expected, livers from AKT/HSF1dn mice were the only ones to express the transfected HSF1dn construct (V5-tagged). Of note, the levels of some of the proteins tested were even lower in AKT/HSF1dn mice than in control mice. Ten livers from each group of mice were used for the analysis, and representative images are shown. β-Actin was used as a loading control. (**C**–**F**) Fatty acid biosynthesis, cholesterol and triglyceride content, and lactate dehydrogenase activity were strongly induced in the livers of AKT mice, and equally lower in control and AKT/HSF1dn livers. Tukey–Kramer test: *P* < 0.0001 *a*, vs control (mice injected with empty vector); *b*, vs AKT mice. Abbreviations: FA, fatty acid; CE, cholesterol; TG, triglycerides; LDH, lactate dehydrogenase.

Subsequently, we determined the importance of HSF1 on HCC growth *in vivo*, focusing on the PI3K/AKT/mTOR pathway. For this purpose, we overexpressed the activated form of *AKT1* (*myr-AKT1*) [25] while simultaneously inactivating HSF1 in the hepatocytes. To achieve this goal, we hydrodynamically transfected FVB/N mice with myr-*AKT1* and a dominant negative form of HSF1 (HSF1dn; these mice will be referred to as AKT/HSF1dn mice; *n* = 10) [26]. As a control, we hydrodynamically transfected FVB/N mice with either empty vector (referred to as control mice; *n* = 10) or myr-*AKT1* (referred to as AKT mice; *n* = 10). Subsequently, mice were harvested 28 weeks post hydrodynamic injection, when livers overexpressing *myr-AKT1* exhibited tumor development [25]. Macroscopically, all AKT mice showed an enlarged liver with a consequent increased body and liver weight when compared to control mice (Figure 4A–4C). In striking contrast, none of the AKT/HSF1dn mice exhibited abdomen enlargement (not shown), increased body or liver weight when compared with control mice (Figure 4B, 4C). Histologically, most of the liver parenchyma of AKT mice was occupied by multiple hepatocellular tumors (Figure 4A), in accordance with previous reports [25]. In striking contrast, none of the AKT/HSF1dn mice showed the presence of neoplastic lesions (Figure 4A). Small clusters of lipid-rich cells were detected in otherwise normal appearing livers in AKT/HSF1dn mice (Figure 4A). At the molecular level, AKT/HSF1dn mice showed downregulation of PIK3CA, p-AKT, p-RPS6, FASN, ACAC, SCD1, PPARγ, ALDOA, and LDHA/C (Figure 5A, 5B). To further confirm the effects of HSF1 silencing on AKT-overexpressing livers, we assayed the levels of fatty acid biosynthesis, cholesterol and triglycerides levels in livers from control, AKT, and AKT/HSF1dn livers at this time point. As expected, AKT liver specimens showed the highest levels of fatty acid biosynthesis, cholesterol and triglycerides, whereas similar levels were detected in control and AKT/HSF1dn mice (Figure 5C–5E). In addition, levels of glycolysis, as assessed by LDH activity, were highest in AKT mice and equivalent in control and AKT/HSF1dn mice (Figure 5F).

Altogether, the present data indicate that HSF1 inactivation is detrimental for AKT-induced hepatocarcinogenesis.

### HSF1 is an independent prognostic indicator in human HCC patients

**Figure 6:**
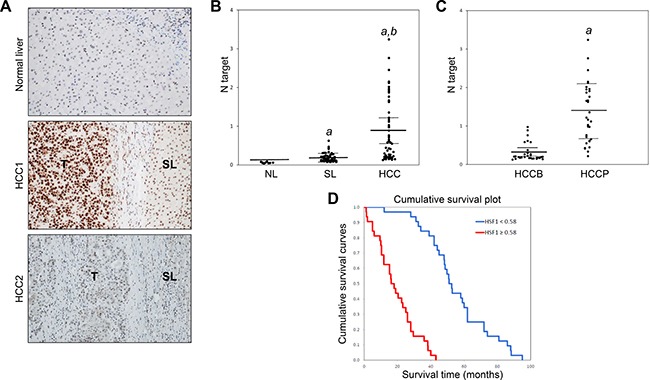
Elevated levels of HSF1 in human hepatocellular carcinoma (HCC) (**A**) Representative immunohistochemistry of HSF1 in human normal liver and HCC. In normal liver (upper panel), nuclear immunoreactivity for HSF1 is either faint or absent. In human HCC (HCC1; T), immunolabeling for nuclear HSF1 was most often higher than that in corresponding non-tumorous surrounding livers (SL) (middle panel). However, few cases showed equivalent staining for HSF1 in HCC (HCC2) and respective SL (lower panel). Original magnification: 100 ×. (**B**) Levels of HSF1 mRNA progressively increase from normal livers (NL; *n* = 10) to non-tumorous surrounding livers (SL; *n* = 64) to HCC (*n* = 64), as detected by quantitative reverse-transcription PCR. Number target (NT) = 2^−ΔCt^, wherein ΔCt value of each sample was calculated by subtracting the average Ct value of the HSF1 gene from the average Ct value of the β-actin gene. Mann–Whitney test: *a, vs* NL, *P* < 0.001; *b: vs* SL, *P* < 0.0001. (**C**) Importantly, levels of HSF1 mRNA are significantly higher in HCC with poorer outcome (HCCP) than in tumors with better prognosis (HCCB). Mann–Whitney test: *a vs* HCCB, *P* < 0.001. (**D**) Kaplan–Meier survival curves of human HCC with high and low HSF1 mRNA levels, showing the unfavorable outcome of patients with high HSF1 mRNA expression.

Finally, we evaluated the levels of activated HSF1 in a collection of human HCC specimens (*n* = 120). As a surrogate marker of HSF1 activation, nuclear accumulation of HSF1 protein was investigated by immunohistochemistry (Figure 6). In accordance with previous data [20, 21], nuclear immunoreactivity for HSF1 was very faint or absent in normal liver (Figure 6A, upper panel). Importantly, HSF1 nuclear accumulation was almost ubiquitously (109/120; 90.8%) more pronounced in HCC than in corresponding non-tumorous liver tissues (Figure 6A, middle panel). In the remaining (11/120, 9.2%) HCC samples and respective non-neoplastic liver tissues, HSF1 nuclear accumulation were equivalent (Figure 6A, lower panel). Subsequently, we evaluated the mRNA levels of HSF1 in the HCC samples whose clinicopathological data were available (*n* = 64; Supplementary Table 1). Once again, we found that HSF1 mRNA levels were significantly higher in HCC than in corresponding non-tumorous livers and normal livers (Figure 6B). Furthermore, HSF1 mRNA expression was significantly more elevated in HCC samples with poorer prognosis when compared with those with better prognosis (Figure 6C). When evaluating the relationship between HSF1 and patients’ clinicopathological data, we found that higher expression of HSF1 correlates with lower HCC survival rate (Figure 6D) and this association remains strongly significant after multivariate Cox regression analysis (*P* < 0.0001; Supplementary Table 2), indicating that HSF1 is an independent prognostic factor for HCC. No relationship between the mRNA levels of HSF1 and other clinicopathological features of the patients, including age, gender, etiology, presence of cirrhosis, tumor size, and tumor grade were detected (Supplementary Table 2).

Furthermore, since it has been shown that HSF1 and the AKT/mTOR cascade functionally interacts in other cancer types [12, 13, 15, 27–29], we determined the levels of AKT and mTOR activity and correlated them with HSF1 activity in human HCC specimens (*n* = 64). Of note, the activity of AKT and mTOR was significantly higher in HCC with poorer prognosis when compared with those with better prognosis (Supplementary Figure 2). Furthermore, a strong, direct correlation was detected between HSF1 activity and that of AKT and mTOR (Supplementary Figure 2).

Altogether, the present data indicate that HSF1 is an important prognostic indicator and its activity correlates with the activity of AKT/mTOR cascade in human HCC.

## DISCUSSION

Unconstrained activation of the HSF1 transcription factor has been detected in multiple tumor types, where it exerts pro-growth and survival functions [11–17]. In the present study, we show that HSF1 is aberrantly expressed and possesses oncogenic properties in human HCC. Indeed, we found a significant upregulation of HSF1 in HCC when compared with normal livers and non-neoplastic surrounding livers. These findings suggest that induction of HSF1 is involved in liver malignant transformation. Furthermore, we detected a pronounced increase of HSF1 levels in HCCP when compared with HCCB, thus implying an important role of HSF1 also in HCC biological aggressiveness and patient's outcome. In accordance with the latter findings, levels of HSF1 have been found to directly correlate with an adverse prognosis and tumor recurrence in various cancer entities, including HCC [11–17, 20, 21, 23]. Therefore, the present data strongly support an important prognostic role of HSF1 in human liver cancer.

**Figure 7:**
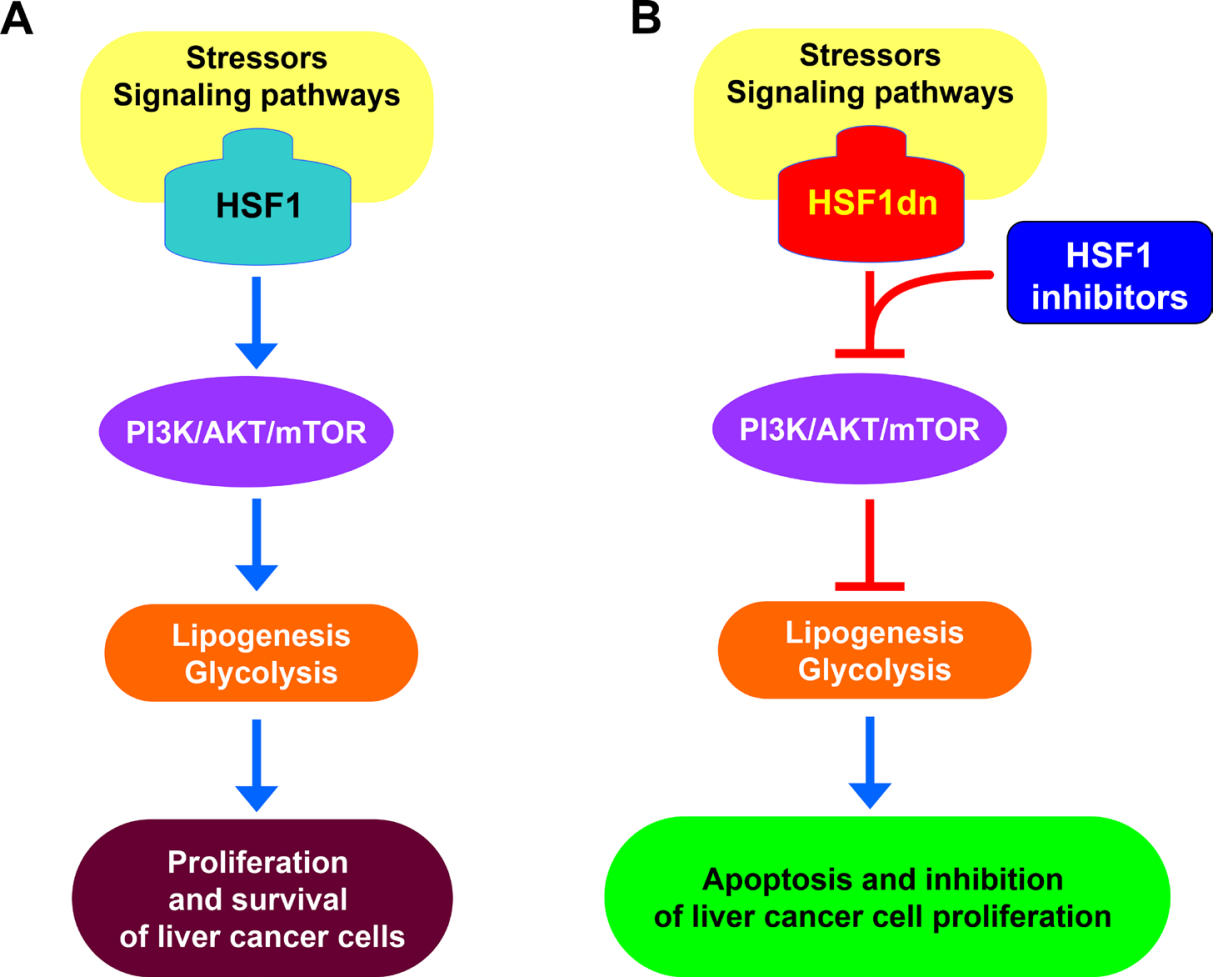
Schematic representation of the relationship between HSF1 and the PI3K/AKT/mTOR pathway in hepatocellular carcinoma (**A**) Following a variety of stimuli that remain to be better delineated (stressors? Signaling pathways?), HSF1 is induced in liver cancer cells, where it sustains the activation of the PI3K/AKT/mTOR cascade. This leads to metabolic reprogramming of liver cancer cells, due to the induction of *de novo* lipogenesis and glycolysis, which support their unconstrained proliferation and survival. (**B**) Inactivation of HSF1 by overexpression of a dominant negative form (HSF1dn) or specific inhibitors results in the suppression of the PI3K/AKT/mTOR pathway, thus inhibiting aberrant lipogenesis and glycolysis with consequent apoptosis and growth restraint of liver cancer cells.

In addition, we have investigated the functional role of HSF1 *in vitro* and *in vivo*. In particular, we found that HSF1 plays a pivotal function in supporting hepatocarcinogenesis induced by the AKT protooncogene, a crucial member of the PI3K/AKT/mTOR pathway (Figure 7). This assumption is based on the inhibition of development of preneoplastic and neoplastic liver lesions in AKT mice where HSF1 was inhibited. Our data, together with the finding of absence of liver malignant transformation in mice hydrodynamically injected with *HSF1* (Calvisi DF et al. unpublished observation) indicate the importance of HSF1 in supporting the oncogenic action driven by protooncogenes rather than acting as a *bona fide* oncogene. These findings agree with the observation that immortalized mouse embryonic fibroblasts (MEFs) cannot be transformed by overexpressing HSF1 alone [17].

The molecular mechanisms whereby HSF1 sustains AKT oncogenic potential and the central players in this event remain to be determined. Nonetheless, we found that several proteins involved in various steps of *de novo* lipogenesis and glycolysis are downregulated in HCC cell lines and in AKT mice following inactivation of HSF1. While the role of glycolysis on AKT-dependent HCC development requires further investigation, the importance of lipogenesis in AKT-driven hepatocarcinogenesis has been underlined by a previous report from our group, in which depletion of FASN, the master regulator of fatty acid biosynthesis, completely abolished AKT-induced HCC development [30, 31]. Besides these effects on the metabolism of HCC cells and AKT-overexpressing livers, we cannot exclude that HSF1 regulates many other important molecular events. For instance, preliminary data from our group suggest that HSF1 inactivation results in the downregulation of the c-Myc protooncogene in HCC cell lines and AKT mice (Calvisi DF et al., unpublished observation). Ongoing studies will be helpful to identify the major targets of HSF1 in AKT-induced hepatocarcinogenesis.

The present data might have important clinical implications. Indeed, the dependence of *AKT*-overexpressing cells on HSF1 activity might render these cells susceptible to death induced by HSF1 inhibitors. In this regard, several HSF1 blockers have been developed and showed remarkable anti-tumor activity in numerous preclinical models [28, 32]. In particular, a class of translation initiation inhibitors, known as rocaglates, has been proven to be extremely effective in suppressing HSF1 activity and to strongly constrain the growth of multiple cancer cell lines [33]. Thus, it is highly likely that HSF1 inhibitors are effective in the treatment of at least the human subset of HCC displaying activation of the AKT pathway.

## MATERIALS AND METHODS

### Constructs and reagents

The constructs used in the experiments, including pT3-EF1α-myr-AKT (HA-tagged) and pCMV/sleeping beauty transposase (SB), have been previously described [25]. The V5-tagged dominant negative form of human HSF1 (HSF1dn) [28] was cloned in a pT3-EF1α vector via Gateway cloning strategy. Plasmids were purified using the Endotoxin-free Maxi Prep Kit (Sigma-Aldrich, St. Louis, MO) before being injected into mice.

### Hydrodynamic injection, mouse monitoring

Hydrodynamic injection was performed as described previously [25]. In brief, 8μg pT3-EF1α- myr-AKT1 along with sleeping beauty transposase (SB) in a ratio of 25:1 were diluted in 2 ml saline (0.9% NaCl), then filtered through 0.22 μm filter, and injected into the lateral tail vein of 6 to 8-week-old FVB/N mice in 5 to 7 seconds. To block the HSF1 signaling, high doses of HSF1dn (40 μg) with low doses of myr-AKT1 (8 μg) were injected. Control mice were injected with pT3-EF1α (40 μg) with myr-AKT1 (8 μg). The care and use of mice for this study were carried out with the approval of the Institutional Animal Care and Use Committee (IACUC) of the University of California, San Francisco.

### Histology and immunohistochemistry

Liver lesions were fixed in 4% paraformaldehyde overnight at 4°C, embedded in paraffin, and evaluated by two board-certified pathologists (M.E. and F.D.) in accordance with the criteria by Frith et al. [34]. For immunohistochemistry, antigen retrieval was performed in 10mM sodium citrate buffer (pH 6.0) by placement in a microwave on high for 10 min, followed by a 20-min cool down at room temperature. After a blocking step with the 5% goat serum and Avidin-Biotin blocking kit (Vector Laboratories, Burlingame, CA), the slides were incubated with primary antibodies overnight at 4°C. Slides were then subjected to 3% hydrogen peroxide for 10 min to quench endogenous peroxidase activity and subsequently the biotin conjugated secondary antibody was applied at a 1:500 dilution for 30 min at room temperature. The anti-HSF1 (# 4356) antibody was obtained from Cell Signaling Technology Inc (Danvers, MA). This primary antibody was selected since it has been extensively validated by the manufacturer for immunohistochemistry. The immunoreactivity was visualized with the Vectastain Elite ABC kit (Vector Laboratories, Burlingame, CA), using Vector NovaRED^™^ (Vector Laboratories) as the chromogen. Slides were counterstained with Mayer's hematoxylin. Immunohistochemical expression of the HSF1 protein was evaluated semi-quantitatively by direct comparison of the tumor area (HCC) with the adjacent non-neoplastic liver parenchyma. Specifically, the HSF1 staining scores were determined as follows: (a.) equivalent, when the immunoreactivity was similar between HCC tissues and corresponding surrounding non-tumorous liver tissues; (b.) overexpression, when immunoreactivity intensity was higher in HCC tissues than in corresponding surrounding non-tumorous liver tissues.

### Western blotting

Human HCC cell line extracts and mouse livers tissues were homogenized in lysis buffer [30 mM Tris (pH 7.5), 150 mM NaCl, 1% NP-40, 0.5% Na deoxycholate, 0.1% SDS, 10% glycerol and 2 mM EDTA] containing the Complete Protease Inhibitor Cocktail (ThermoFisher Scientific, Waltham, MA). Protein concentrations were determined with the Bio-Rad Protein Assay Kit (Bio-Rad, Hercules, CA) using bovine serum albumin as standard. For Western blotting, aliquots of 40 μg were denatured by boiling in Tris-Glycine SDS Sample Buffer (Bio-Rad), separated by SDS-PAGE, and transferred onto nitrocellulose membranes (Bio-Rad) by electroblotting. Membranes were blocked in Pierce Protein-free Tween 20 Blocking Buffer (ThermoFisher Scientific) for 1 h and probed with following specific antibodies for anti-HSF1 (#4356), anti-HA-tag (#3724), anti-phosphorylated AKT (#3787), anti-phosphorylated RPS6 (#2211), anti-SCD1 (#2438), total-AKT (#9272), anti-ACAC (#3662), anti-ACLY (13390), anti-PPARγ (#2435), anti-ALDOA (#3188), LDHA/C (#3558), SCD1 (#2794), anti-phosphorylated NDRG1 (#5482) (Cell Signaling Technology Inc.), and V5 (#81594; Santa Cruz Biotechnology, Santa Cruz, CA). Anti-β-Actin (#A5441; Sigma-Aldrich) was used as loading control. Each primary antibody was followed by incubation with horseradish peroxidase-secondary antibody (Jackson ImmunoResearch Laboratories Inc., West Grove, PA) diluted 1:5000 for 30 min and proteins were revealed with the Super Signal West Pico (Pierce Chemical Co., New York, NY).

### *In vitro* studies

The human HLE and HLF HCC cell lines, after validation (Genetica DNA Laboratories, Burlington, NC), were used in this study. Cells were grown in a 5% CO_2_ atmosphere, at 37°C, in RPMI Medium supplemented with 10% fetal bovine serum (FBS; Gibco, Grand Island, NY) and penicillin/streptomycin (Gibco). Cell proliferation was analyzed using the BrdU Cell Proliferation Assay Kit (Cell Signaling Technology Inc.). Apoptosis was assessed with the Cell Death Detection Elisa Plus Kit (Roche Molecular Biochemicals, Indianapolis, IN).

### Knockdown of HSF1

For knockdown studies, HLE and HLF cells were transfected with 50 nM siRNA targeting *HSF1* (ID # L-012109-02-0005; GE, Dharmacon, Lafayette, CO). A scramble small interfering RNA (siRNA; ID # 4390846; Life Technologies, Grand Island, NY) was used as negative control. RNA was extracted 48 hours after siRNA transfection. Experiments were repeated at least three times in triplicate.

### Quantitative reverse transcription real-time polymerase chain reaction (qRT-PCR)

Validated Gene Expression Assays for human *HSF1* (Hs00232134_m1) and β*- Actin* (ID: 4333762T) genes were purchased from Applied Biosystems (Foster City, CA). PCR reactions were performed with 100 ng of cDNA of the collected samples or cell lines, using an ABI Prism 7000 Sequence Detection System with TaqMan Universal PCR Master Mix (Applied Biosystems). Cycling conditions were: denaturation at 95°C for 10 min, 40 cycles at 95°C for 15 s, and then extension at 60°C for 1 min. Quantitative values were calculated by using the PE Biosystems Analysis software and expressed as N target (NT). NT = 2^−ΔCt^, wherein ΔCt value of each sample was calculated by subtracting the average Ct value of the target gene from the average Ct value of the β*- Actin* gene.

### Assessment of AKT, mTOR, and HSF1 activity

The activity of AKT, mTOR, and HSF1 was determined in human HCC specimens using the PathScan^®^ Phospho-Akt (Ser^473^), and PathScan^®^ Phospho-mTOR (Ser^2448^) Sandwich ELISA Kits (Cell Signaling Technology), and (pSer^326^) HSF1 ELISA kit (Enzo Life Sciences, Farmingdale, NY), respectively, following the manufacturers’ instructions.

### Assessment of cholesterol and triglyceride content, and lactate dehydrogenase activity

Fatty acid synthesis was measured in mouse frozen tissues and human HCC cell lines by incorporation of [U-^14^C] acetate into lipids as described [35]. Briefly, HCC cell lines were cultured in 96-well plates at 2.0 × 10^3^/well and incubated overnight. After adding specific or scramble siRNA, cells were pulse labelled with [U-^14^C] acetate (1 μCi/well) for 48 hours. Each condition was run in triplicate. Lipids were Folch extracted and counted for ^14^C. Cholesterol and triglyceride levels in HCC cell lines and mouse specimens were assessed using the Cholesterol Quantitation Kit and the Triglyceride Quantification Kit (BioVision Inc., Mountain View, CA), respectively, following the manufacturer's protocol. Lactate dehydrogenase activity was assessed using the LDH activity assay (BioVision Inc.) according to the manufacturer's instructions.

### Human tissue samples

A collection of formalin-fixed, paraffin-embedded HCC samples was used in the present study. Sixty-four frozen HCC and corresponding non-tumorous surrounding livers from the same collection were used. Tumors were divided in HCC with shorter/poorer (HCCP; *n* = 32) and longer/better (HCCB; *n* = 32) survival, characterized by < 3 and > 3 years’ survival following partial liver resection, respectively. The clinicopathological features of liver cancer patients are summarized in Supplementary Table 1. HCC specimens were collected at the Medical Universities of Greifswald (Greifswald, Germany) and Rome La Sapienza (Pietro Valdoni Surgery Department, Rome, Italy). Institutional Review Board approval was obtained at the local Ethical Committee of the Medical Universities of Greifswald and Rome. Informed consent was obtained from all individuals.

### Statistical analysis

GraphPad Prism version 6.0 (GraphPad Software Inc., La Jolla, CA) was used to evaluate statistical significance by Tukey–Kramer, Student's *t* and Mann–Whitney tests and linear regression analyses. Overall survival was estimated according to Kaplan–Meier and Log-rank (Mantel–Cox) test. Univariate and multivariate Cox analysis was used to estimate hazard ratios for risk factors using STATA 9 statistical software (Stata Corporation, College Station, TX, USA). Values of *P* < 0.05 were considered significant. Data are expressed as mean ± standard deviation for each group. Two-tailed unpaired *t* test was used to compare the differences between two groups.

## SUPPLEMENTARY MATERIALS FIGURES AND TABLES



## Abbreviations

ACAC: acetyl-CoA carboxylase
ACLY: ATP citrate lyase
AKT: v-Akt murine thymoma viral oncogene homolog
CE: cholesterol
FASN: fatty acid synthase
HCC: hepatocellular carcinoma
HCCB: hepatocellular carcinoma with better/longer survival
HCCP: hepatocellular carcinoma with poorer/shorter survival
HSF1: heat shock transcription factor 1
HSP: heat shock protein
LDH: lactate dehydrogenase
mTOR: mammalian target of rapamycin
mTORC: mTOR complex
PI3K: phosphoinositide 3-kinase
PIK3CA: phosphatidylinositol-4,5-bisphosphate 3-kinase catalytic subunit alpha
PKM1/2: pyruvate kinase M1/2
RPS6: ribosomal protein S6
SCD1: stearoyl-CoA desaturase 1
siRNA: small interfering RNA
TG: triglycerides.

## References

[1] Pascual S, Herrera I, Irurzun J (2016). New advances in hepatocellular carcinoma. World J Hepatol.

[2] El-Serag HB (2011). Hepatocellular carcinoma. N Engl J Med.

[3] Llovet JM, Ricci S, Mazzaferro V, Hilgard P, Gane E, Blanc JF, de Oliveira AC, Santoro A, Raoul JL, Forner A, Schwartz M, Porta C, Zeuzem S (2008). Sorafenib in advanced hepatocellular carcinoma. N Engl J Med.

[4] Sorger PK (1991). Heat shock factor and the heat shock response. Cell.

[5] Young JC, Agashe VR, Siegers K, Hartl FU (2004). Pathways of chaperone-mediated protein folding in the cytosol. Nat Rev Mol Cell Biol.

[6] Westerheide SD, Morimoto RI (2005). Heat shock response modulators as therapeutic tools for diseases of protein conformation. J Biol Chem.

[7] Pirkkala L, Nykanen P, Sistonen L (2001). Roles of the heat-shock transcription factors in regulation of the heat-shock response and beyond. FASEB J.

[8] Voellmy R (1994). Transduction of the stress signal and mechanisms of transcriptional regulation of heat shock/stress protein gene expression in higher eukaryotes. Crit Rev Eukaryot Gene Exp.

[9] Soncin F, Prevelige R, Calderwood SK (1997). Expression and purification of human heat-shock transcription factor 1. Protein Expr Purif.

[10] Hahn JS, Hu Z, Thiele DJ, Iyer VR (2004). Genome-wide analysis of the biology of stress responses through heat shock transcription factor. Mol Cell Biol.

[11] Mendillo ML, Santagata S, Koeva M, Bell GW, Hu R, Tamimi RM, Fraenkel E, Ince TA, Whitesell L, Lindquist S (2012). HSF1 drives a transcriptional program distinct from heat shock to support highly malignant human cancers. Cell.

[12] Home T, Jensen RA, Rao R (2015). Heat shock factor 1 in protein homeostasis and oncogenic signal integration. Cancer Res.

[13] Jiang S, Tu K, Fu Q, Schmitt DC, Zhou L, Lu N, Zhao Y (2015). Multifaceted roles of HSF1 in cancer. Tumour Biol.

[14] Vihervaara A, Sistonen L (2014). HSF1 at a glance. J Cell Sci.

[15] Calderwood SK, Khaleque MA, Sawyer DB, Ciocca DR (2006). Heat shock proteins in cancer: chaperones of tumorigenesis. Trends Biochem Sci.

[16] Jolly C, Morimoto RI (2000). Role of the heat shock response and molecular chaperones in oncogenesis and cell death. J Natl Cancer Inst.

[17] Chengkai Dai, Luke Whitesell, Rogers AB, Lindquist S (2007). Heat Shock Factor 1 Is a Powerful Multifaceted Modifier of Carcinogenesis. Cell.

[18] Min JN, Huang L, Zimonjic DB, Moskophidis D, Mivechi NF (2007). Selective suppression of lymphomas by functional loss of Hsf1 in a p53-deficient mouse model for spontaneous tumors. Oncogene.

[19] Jin X, Moskophidis D, Mivechi NF (2011). Heat shock transcription factor 1 is a key determinant of HCC development by regulating hepatic steatosis and metabolic syndrome. Cell Metab.

[20] Fang F, Chang R, Yang L (2012). Heat shock factor 1 promotes invasion and metastasis of hepatocellular carcinoma *in vitro* and *in vivo*. Cancer.

[21] Li S, Ma W, Fei T, Lou Q, Zhang Y, Cui X, Qin X, Zhang J, Liu G, Dong Z, Ma Y, Song Z, Hu Y (2014). Upregulation of heat shock factor 1 transcription activity is associated with hepatocellular carcinoma progression. Mol Med Rep.

[22] Liu D, Sun L, Qin X, Liu T, Zhang S, Liu Y, Li S, Guo K (2016). HSF1 promotes the inhibition of EMT-associated migration by low glucose via directly regulating Snail1 expression in HCC cells. Discov Med.

[23] Evert M, Frau M, Tomasi ML, Latte G, Simile MM, Seddaiu MA, Zimmermann A, Ladu S, Staniscia T, Brozzetti S, Solinas G, Dombrowski F, Feo F (2013). Deregulation of DNA-dependent protein kinase catalytic subunit contributes to human hepatocarcinogenesis development and has a putative prognostic value. Br J Cancer.

[24] Ma W, Zhang Y, Mu H, Qing X, Li S, Cui X, Lou Q, Ma Y, Pu H, Hu Y (2015). Glucose regulates heat shock factor 1 transcription activity via mTOR pathway in HCC cell lines. Cell Biol Int.

[25] Calvisi DF, Wang C, Ho C, Ladu S, Lee SA, Mattu S, Destefanis G, Delogu S, Zimmermann A, Ericsson J, Brozzetti S, Staniscia T, Chen X (2011). Increased lipogenesis, induced by AKT-mTORC1-RPS6 signaling, promotes development of human hepatocellular carcinoma. Gastroenterology.

[26] Wang Y, Theriault JR, He H, Gong J, Calderwood SK (2004). Expression of a dominant negative heat shock factor-1 construct inhibits aneuploidy in prostate carcinoma cells. J Biol Chem.

[27] Chou SD, Prince T, Gong J, Calderwood SK (2012). mTOR is essential for the proteotoxic stress response, HSF1 activation and heat shock protein synthesis. PLoS One.

[28] McConnell JR, Buckton LK, McAlpine SR (2015). Regulating the master regulator: Controlling heat shock factor 1 as a chemotherapy approach. Bioorg Med Chem Lett.

[29] Dayalan Naidu S, Dinkova-Kostova AT (2017). Regulation of the mammalian heat shock factor 1. FEBS J.

[30] Li L, Pilo GM, Li X, Cigliano A, Latte G, Che L, Joseph C, Mela M, Wang C, Jiang L, Ribback S, Simile MM, Pascale RM (2016). Inactivation of fatty acid synthase impairs hepatocarcinogenesis driven by AKT in mice and humans. J Hepatol.

[31] Che L, Pilo MG, Cigliano A, Latte G, Simile MM, Ribback S, Dombrowski F, Evert M, Chen X, Calvisi DF (2017). Oncogene dependent requirement of fatty acid synthase in hepatocellular carcinoma. Cell Cycle.

[32] Yoon YJ, Kim JA, Shin KD, Shin DS, Han YM, Lee YJ, Lee JS, Kwon BM, Han DC (2011). KRIBB11 inhibits HSP70 synthesis through inhibition of heat shock factor 1 function by impairing the recruitment of positive transcription elongation factor b to the hsp70 promoter. J Biol Chem.

[33] Santagata S, Mendillo ML, Tang YC, Subramanian A, Perley CC, Roche SP, Wong B, Narayan R, Kwon H, Koeva M, Amon A, Golub TR, Porco JA (2013). Tight coordination of protein translation and HSF1 activation supports the anabolic malignant state. Science.

[34] Frith CH, Ward JM, Turusov VS (1994). Tumours of the liver. IARC Sci Publ.

[35] Pizer ES, Thupari J, Han WF, Pinn ML, Chrest FJ, Frehywot GL, Townsend CA, Kuhajda FP (2000). Malonyl-coenzyme-A is a potential mediator of cytotoxicity induced by fatty-acid synthase inhibition in human breast cancer cells and xenografts. Cancer Res.

