# Supplementary material for: Inhibition of HSF1 suppresses the growth of hepatocarcinoma cell lines in vitro and AKT-driven hepatocarcinogenesis in mice
Source: Oncotarget. 2017 Apr 7;8(33):54149–59. doi: 10.18632/oncotarget.16927 (PMC5589569; doi:10.18632/oncotarget.16927)
Supplement: Supplementary file 1 [file oncotarget-08-54149-s001.pdf]

Inhibition of HSF1 suppresses the growth of hepatocarcinoma cell lines *in vitro* and AKT-driven hepatocarcinogenesis in mice

Supplementary Materials

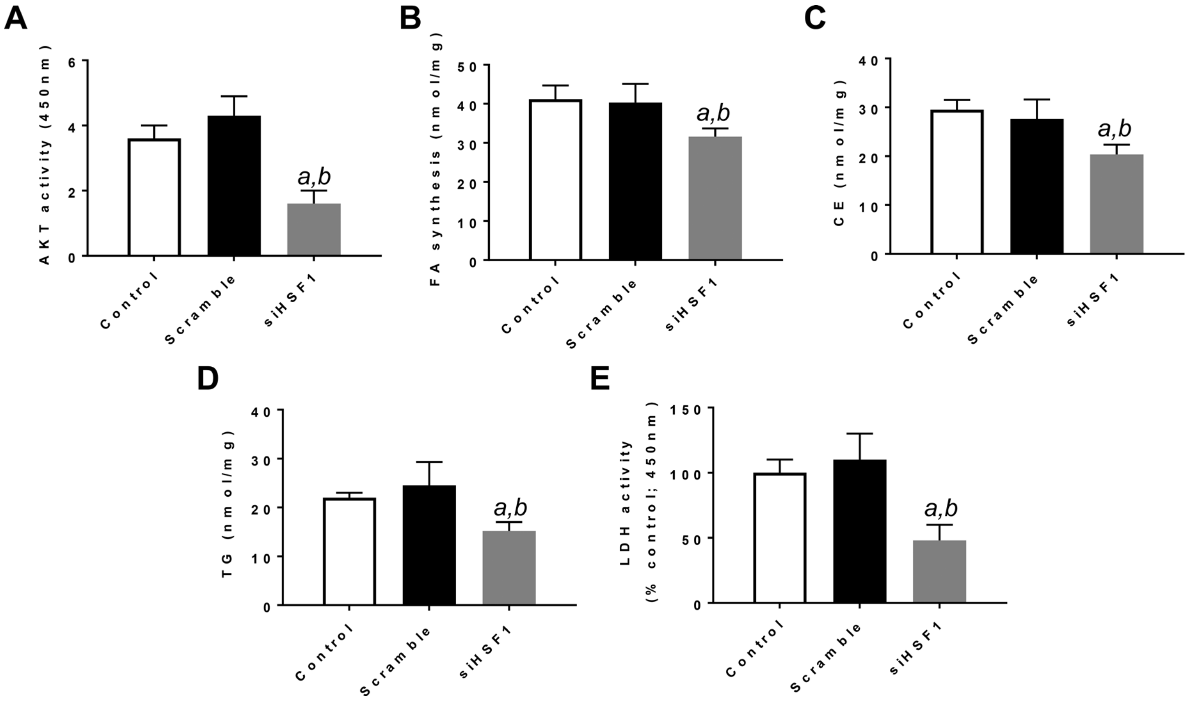

Supplementary Figure 1: Suppression of *HSF1* expression by specific siRNA induces decrease in AKT activity, fatty acid biosynthesis, cholesterol and triglyceride levels, and lactate dehydrogenase activity in the HLF HCC cell line. Each *bar* represents mean ± standard deviation of 3 independent experiments conducted in triplicate. Tukey–Kramer test: $P < 0.0001$ *a*, vs control (untreated cells); *b*, vs scramble siRNA. Abbreviations: FA, fatty acid; CE, cholesterol; TG, triglycerides; LDH, lactate dehydrogenase.

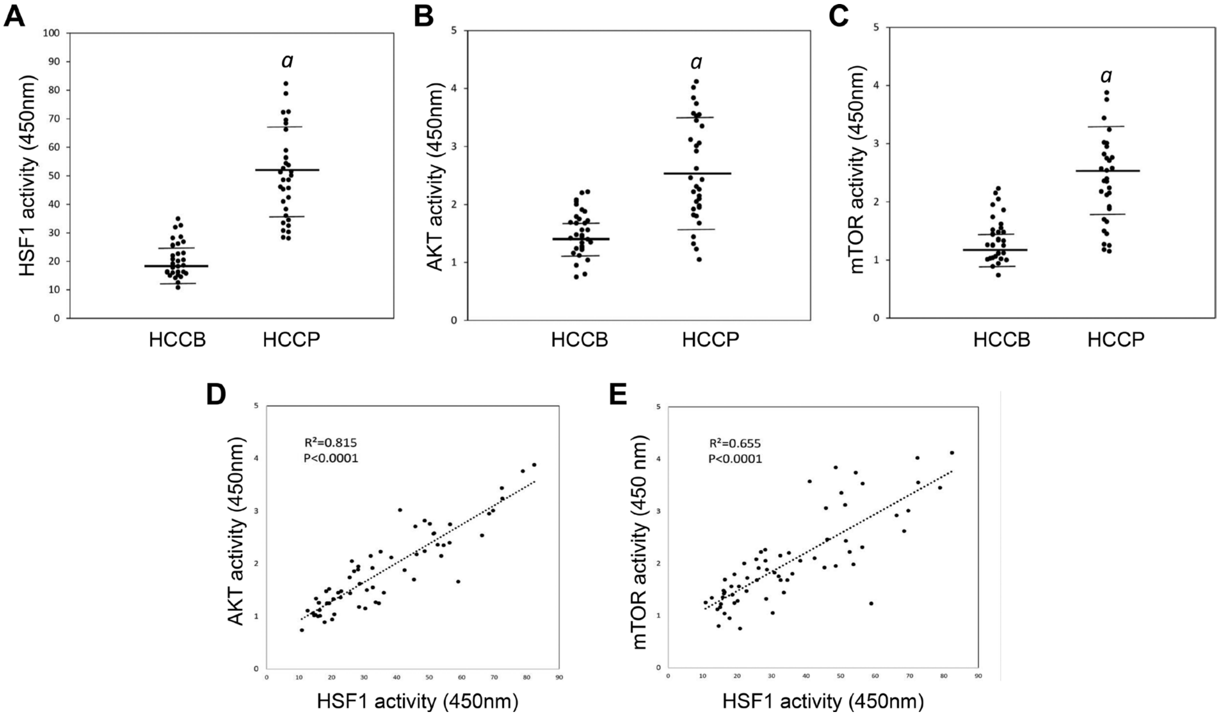

Supplementary Figure 2: Activity of HSF1, AKT, and mTOR is associated with an adverse prognosis in human HCC. A total of 64 human HCC specimens (32 HCC with better prognosis or HCCB and 32 with poorer prognosis or HCCP) were analyzed for the activity of HSF1 (**A**), AKT (**B**), mTOR (**C**) using commercially available ELISA kits following the manufacturers‘ instructions. Of note, a significant, direct correlation was detected between the activity of HSF1 and that of AKT (**D**) and mTOR (**E**) in the same specimens. Student's *t* test: $P < 0.0001$ a, vs HCCB.

Supplementary Table 1: Clinicopathological features of HCC patients

Variables	Features	
	**HCCB[a]**	**HCCP[b]**
No. of patients	32	32
Male	26	25
Female	6	7
Age (Mean ± SD)	62.8 ± 10.2	65.5 ± 12.0
Etiology		
HBV	16	14
HCV	11	11
Ethanol	2	4
Wilson's disease	1	2
Hemochromatosis	1	1
Cirrhosis		
+	22	24
−	10	8
Tumor size		
> 5 cm	20	23
< 5 cm	12	9
Edmondson and Steiner grade		
II	10	10
III	14	11
IV	8	11
Alpha-fetoprotein secretion		
> 300 ng/ml of serum	20	20
< 300 ng/ml of serum	12	12
Survival after partial liver resection		
(months)	58.6	20.2
Means ± SD	± 18.2	± 10.2

[a]HCCb, HCC with better outcome/longer survival (survival > 3 years following partial liver resection).
[b]HCCp, HCC with poorer outcome/shorter survival (survival < 3 years following partial liver resection).

Supplementary Table 2: Multivariate Cox regression analysis of factors contributing to overall survival of HCC patients

Covariates	Full model (HR and 95% CI)	Stepwise backward elimination (HR and 95% CI)
Age	1.018 (0.990–1.047)	–
Male sex	0.661 (0.337–1.298)	0.613 (0.341–1.103)
Cirrhosis (y/n)	0.696 (0.353–1.373)	–
Etiology		
HCV	Reference	–
HBV	1.220 (0.635–2.344)	
Ethanol	1.105 (0.391–3-117)	
Wilson's disease and/or Hemochromatosis	0.698 (0.253–1.927)	
Diameter > 3 cm	1.416 (0.658–3.046)	–
AFP > 300 ng/ml	1.786 (0.986–3.234)	1.642 (0.940–2.870)
Grade		
II	Reference	–
III	0.813 (0.401–1.650)	
IV	1.246 (0.589–2.637)	
HSF1 > 0.58 (median value)	17.629 (7.207–43.124)**	17.837 (7.482–42.522)**

*$P < 0.05$; **$P < 0.0001$.
